# Green Synthesis, Characterization, and Evaluation of the Antimicrobial Properties and Compressive Strength of Hydroxyapatite Nanoparticle-Incorporated Glass Ionomer Cement

**DOI:** 10.7759/cureus.58562

**Published:** 2024-04-18

**Authors:** Priyan Ilancheran, Jessy Paulraj, Subhabrata Maiti, Rajeshkumar Shanmugam

**Affiliations:** 1 Department of Pedodontics and Preventive Dentistry, Saveetha Dental College and Hospital, Saveetha Institute of Medical and Technical Sciences, Saveetha University, Chennai, IND; 2 Department of Prosthodontics, Saveetha Dental College and Hospital, Saveetha Institute of Medical and Technical Sciences, Saveetha University, Chennai, IND; 3 Nanobiomedicine Lab, Centre for Global Health Research, Saveetha Medical College and Hospital, Saveetha Institute of Medical and Technical Sciences, Chennai, IND

**Keywords:** compressive strength, modified gic, antimicrobial activity, green synthesis, hydroxyapatite nanoparticle

## Abstract

Background

Glass ionomer cement (GIC) plays a vital role in dental restorative procedures, serving purposes such as filling, luting, and adhesion. However, its inadequate mechanical properties pose challenges, especially in areas experiencing significant stress. To overcome this limitation, nanohydroxyapatite (nHA), known for its bioactive phosphate content, is added to the GIC at specific concentrations to improve its properties.

Aim

We aim to evaluate the antimicrobial property and compressive strength of green-mediated nHA-incorporated GIC.

Material and methods

Green synthesis of hydroxyapatite nanoparticles was prepared using *Moringa oleifera* extract in a solvent form and eggshell waste served as the calcium source. These nHA powders were then integrated into the GIC at varying concentrations (3%, 5%, and 10%) designated as Group I, Group II, and Group III, respectively, while Group IV (control) consisted of conventional GIC. Specimens were fabricated and subjected to chemical structure analysis through Fourier transform infrared spectroscopy (FTIR), energy dispersive X-ray (EDX), and scanning electron microscopy (SEM). The antimicrobial activity and compressive strength of all groups were investigated. The antimicrobial activity against *Streptococcus mutans* and *Lactobacillus *was evaluated through the minimum inhibitory concentration (MIC) test, while compressive strength was evaluated by measuring the maximum force endured by the specimen before fracturing. Data analysis utilized IBM SPSS Statistics software, employing repeated measures ANOVA to determine mean MIC values and compressive strength, with Tukey's posthoc test for pairwise comparisons.

Results

The results of the study showed that the antimicrobial efficacy of nHA GIC improved with increasing weight percent (% wt) of the additive, exhibiting significantly enhanced activity against *Streptococcus mutans* and *Lactobacillus* compared to the control group (Group IV) with statistical significance (p < 0.05). Moreover, the compressive strength exhibited notable enhancements in the modified groups, including Group I (172.55 ± 0.76), Group II (178.16 ± 0.760), and Group III (182.45 ± 0.950), when compared to the control (162.46 ± 1.606), with statistically significant differences (p < 0.05).

Conclusion

The study demonstrates that the incorporation of green-mediated nHA-containing GIC results in superior antimicrobial efficacy and compressive strength compared to the control group (Group IV). In particular, the highest concentration of nHA-modified GIC (10%) exhibited the most favorable antimicrobial properties along with increased strength. Therefore, utilizing green-mediated nHA in the GIC shows promise as an effective restorative material. Future investigations should delve into the molecular chemistry and bonding mechanisms to further explore its potential.

## Introduction

The effectiveness of glass ionomer cement (GIC) in dentistry is attributed to its distinctive characteristics, such as excellent adhesion to the teeth even in moist oral environments, obviating the need for additional adhesion-promoting agents [1]. This adhesion capability of the GIC facilitates the sealing of marginal gaps at the tooth-restoration interface [2]. However, despite its advantages, the GIC suffers from drawbacks, particularly its low mechanical strength and susceptibility to fracture, which is attributed to its brittle nature [3,4]. Moreover, the GIC's limited resistance to moisture and desiccation during the setting process, as noted by Arita et al. [5], restricts its application in high-stress areas due to potential dehydration. To mitigate these limitations, researchers have explored the incorporation of additional additives into GIC formulations. In the past few years, the domain of dentistry has seen a rapid integration of nanotechnology, offering novel approaches to address various scientific and medical challenges [6]. Previous research has explored the incorporation of nanoparticles to enhance the mechanical and physical properties of the GIC [1]. While these modified materials have demonstrated improved strength characteristics and reduced wear, they have also presented drawbacks such as decreased strength and potential cytotoxicity, limiting their practical utility [7].

Hydroxyapatite, characterized by Ca10(PO4)6(OH)2, is a notable calcium phosphate ceramic within the apatite family [8]. It can be naturally sourced from biological materials such as eggshells and aquatic organisms like fish bones and seashells [9], providing both environmental and economic benefits. This compound closely resembles the composition of mineralized tissues found in bones and teeth [10,11], earning it the moniker "bone mineral." Hydroxyapatite demonstrates excellent biocompatibility in hard tissues, exhibiting minimal cytotoxic effects [12]. Hydroxyapatite nanoparticles have garnered considerable attention in nanotechnology because of their exceptional purity, distinct structure, and accurate chemical composition [13]. The utilization of green synthesis techniques for producing hydroxyapatite nanoparticles presents an innovative and promising approach within materials science and dentistry. This method encompasses a variety of sustainable and environmentally friendly processes aimed at fabricating hydroxyapatite nanoparticles and evaluating their distinctive characteristics. Green synthesis methods prioritize reducing environmental impact and energy consumption by employing eco-friendly precursors and methodologies, aligning with the increasing emphasis on sustainability. Eggshells, primarily composed of calcium carbonate, offer a viable alternative to limestone-based calcium carbonate and can be effectively utilized in hydroxyapatite synthesis [14]. The calcium carbonate extracted from eggshells acts as a starting material for producing hydroxyapatite nanoparticles. Leveraging plant materials for hydroxyapatite nanoparticle synthesis offers advantages such as simplicity and minimal resource requirements [15]. In this research, we outline the synthesis of hydroxyapatite nanoparticles utilizing calcium oxide obtained from eggshells and facilitated by *Moringa oleifera *leaf extract. *Moringa oleifera* is a widely accessible plant known for its nutritional value, rich iron content, and medicinal properties.

In dentistry, particular attention is given to the antimicrobial properties of nanoparticles within the GIC [16]. These nanoparticles have the potential to hinder the growth of harmful bacteria, offering both restorative and preventive benefits [17]. Such properties are crucial for dental materials, as they can enhance the durability of restorations and promote oral health. Hydroxyapatite, often referred to as "bone mineral," possesses properties favorable for dental restoration. Therefore, this study aims to modify the GIC with nanohydroxyapatite (nHA) and evaluate both its antibacterial efficacy and compressive strength. The null hypothesis assumes that these modifications will not demonstrate antibacterial effects or affect compressive strength compared to conventional GIC.

## Materials and methods

Ethical approval, estimation of sample size, and materials used

Ethical approval was acquired under the code SRB/SDC/UG-2006/23/PEDO/049. Sample size calculation was performed using the GPower sample power calculator, aiming for a sample power of 0.95 (95% confidence interval), and an effect size of 0.6 with each parameter (antimicrobial property and compressive strength) would need to include 48 samples. The GIC utilized in the study consisted of aluminosilicate glasses and polyacids (GC Corporation, Tokyo, Japan). Eggshells for the research were obtained from the institute's cafeteria within the campus in Chennai, India. *Moringa oleifera* leaves were collected from the garden situated within the campus vicinity.

Green synthesis of hydroxyapatite nanoparticles

**Figure 1:**
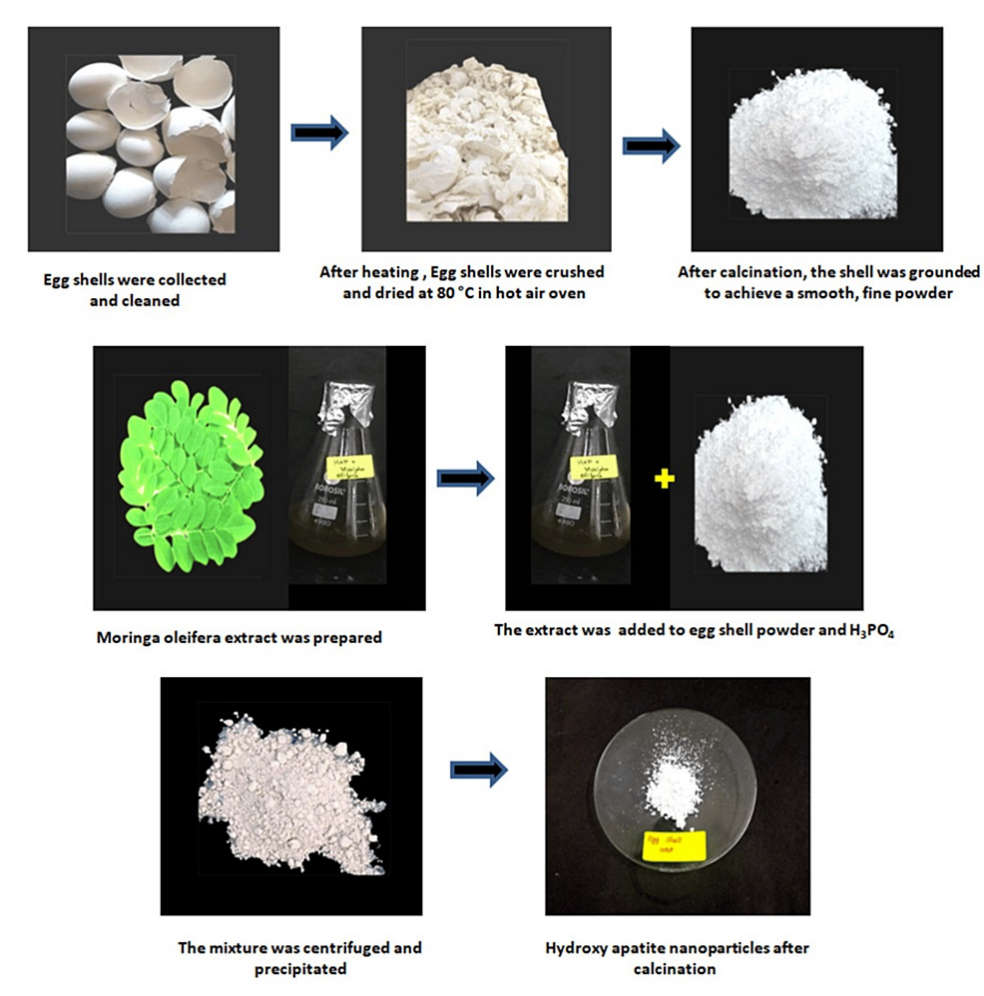
Extraction of green synthesis of hydroxyapatite nanoparticles

The eggshells underwent a rigorous cleaning process involving multiple rinses with tap water and distilled water; after which, they were heated at 80°C in a water bath for half an hour; and the material was then dried for 24 hours at the same temperature in a hot air oven. Following the drying process, they were crushed into powder and exposed to a temperature of 1000°C for two hours in a muffle furnace. After cooling down, the eggshells were finely crushed to create calcium oxide (CaO) powder. The extract of *Moringa oleifera *was prepared. A solution containing CaO in stoichiometric amounts was then prepared in the extract and continuously stirred on a hot plate for two hours. Orthophosphoric acid (Merck Lab Chemicals, India) was added dropwise, stirred thoroughly, and aged for one day. Following centrifugation, the precipitate underwent washing, drying, and then calcined at 900°C to produce white hydroxyapatite nanoparticles (Figure 1).

Incorporation technique of green-mediated nHA into the GIC

The integration of nHA particles into the GIC was carried out by adding powdered nHA at varying weight percentages 3%, 5%, and 10%, respectively. The powdered mixture, consisting of conventional GIC powder and nHA, was placed in Eppendorf tubes and vigorously mixed for one minute using a vortex machine, which is a standard mixing technique [18]. Following this, the powdered blend was combined with the liquid component of the GIC according to the prescribed powder/liquid ratio specified by the manufacturer. A commercially available GIC sourced from GC Corporation, Japan, was used as the control.

Fourier transform infrared (FTIR) analysis

FTIR was utilized to assess the modifications following the addition of hydroxyapatite nanoparticles. FTIR (Nicolet iS10; Thermo Fisher Scientific, Waltham, Massachusetts) was obtained for the GIC nHA powder within the spectral range of 400-4000 cm^-1^, at a resolution of 4 cm^-1^, to analyze the chemical bonds existing in the samples.

Energy dispersive X-ray (EDX) analysis

EDX was employed to determine the elements. Cement samples were prepared in powder form and examined, and the EDX (FEA Company of USA (S.E.A) PTE LTD) findings were quantified in terms of element weight percentages, with two decimal places precision.

Scanning electron microscopy (SEM) analysis

The nHA-dehydrated powder's microstructure was examined using SEM (Jeol, JSM IT-800, Germany). Before the assessment, each powder specimen was coated with gold using a sputter coater at a gas pressure of around 50 mTorr and a current of approximately 40 mA. The coating process lasted for 180 seconds.

Evaluation of antibacterial activity

Circular samples measuring 6 mm in diameter and 2 mm in height were produced for each group. The prepared cement, as per the respective weight percentages, was inserted into divided Teflon molds, covered on top with a celluloid strip, and a glass slab under manual compression. Following a 15-minute setting period at room temperature, the glass ionomer was left undisturbed for 24 hours to ensure a complete setting. Afterward, the disc-shaped samples were removed from the molds by pushing the bottom of the mold upward using finger pressure, followed by the careful removal of excess material with a scalpel. Cultures of *Streptococcus mutans* and *Lactobacillus acidophilus* were obtained from the microbiology laboratory, cultured on Mueller-Hinton agar, and then transferred to Mueller-Hinton broth following a 24-hour incubation period at 37°C. The bacterial suspensions were standardized to a concentration of 1.5 × 10^8^ CFU/ml. To assess antimicrobial efficacy, each group consisted of 12 specimens (six for *Streptococcus mutans* testing and the remaining six for *Lactobacillus*) resulting in a total of 48 specimens. Mueller-Hinton agar broth was prepared and distributed evenly among all wells within the groups. Bacterial suspensions of *Streptococcus mutans* and *Lactobacillus acidophilus *were introduced into the wells. Minimal inhibitory concentration (MIC) assays were conducted individually for each group, including modified specimens with varying weight percentages (3%, 5%, and 10%), as well as unmodified GIC serving as the control. Therefore, the antimicrobial assay was conducted 12 times for each group, in order to ensure robustness, reliability, validity, and adequate replication of the experiment in the assessment of antimicrobial efficacy. Throughout the incubation period, samples were observed for durations (ranging from one to five hours). The percentage of cell death at specific time points was determined using an enzyme-linked immunosorbent assay (ELISA) reader, measuring absorbance at a wavelength of 540 nm.

Compressive strength measurement

Compressive strength evaluation was conducted in compliance with ISO 9917-1:2007 standards. Each group comprised 12 specimens with a total of 48 specimens, fabricated using cylindrical molds (4.0 mm diameter and 6.0 mm height). Before filling the molds, a fine coating of cocoa butter which was supplied by the manufacturer of conventional GIC was applied to aid in the retrieval of set samples. Subsequently, the molds were filled with the material and covered with glass slides, followed by gentle manual pressure to eliminate the entry air bubbles. After 30 minutes, the molds were carefully disassembled, and the specimens were delicately extracted manually and immersed in distilled water at 37°C for 24 hours for conditioning. Each sample's diameter was gauged using a digital micrometer before vertical placement in a universal testing machine (Instron, ElectroPuls, India). Compression force was exerted longitudinally on the specimens at a crosshead speed of 0.5 mm/min until they fractured and the resultant data was recorded. Therefore, the test was conducted 12 times for each group to ensure the reliability, validity, and sufficient replication of the experiment in assessing compressive strength.

Statistical analysis

Mean differences were examined using one-way analysis of variance (ANOVA) followed by Tukey’s posthoc test for statistical significance. This statistical analysis was carried out using the IBM SPSS Statistics for Windows, Version 24 (Released 2016; IBM Corp., Armonk, New York, United States). A significance level of p < 0.05 was set to determine statistical significance.

## Results

FTIR spectroscopy

**Figure 2:**
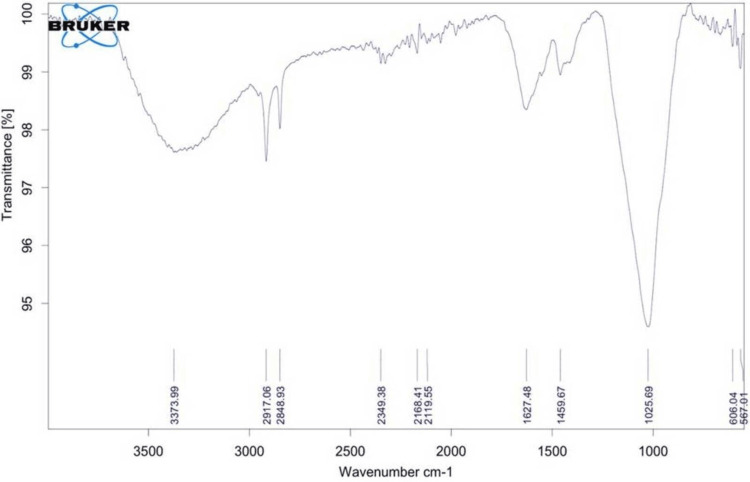
FTIR pattern of nanohydroxyapatite-modified GIC FTIR: Fourier transform infrared spectroscopy; GIC: glass ionomer cement

FTIR analysis was conducted on nHA-modified GIC (Figure 2).

According to the figure, the v2 O-P-O bending mode appeared at around 567 cm^−1^, while the v4 O-P-O bending mode was observed near 606 cm^−1^. Additionally, the FTIR spectral band at approximately 1025 cm^−1^ indicated the vibrational mode of v3 asymmetric P-O stretching. C-O and asymmetric COOH vibration modes were detected at approximately 1459 and 1627 cm^−1^, respectively. Another peak was identified at approximately 2917 cm^−1^, indicating the OH vibration mode, while the OH-F vibration mode was observed at around 3373 cm^−1^. These results suggest the successful embedding of HA nanoparticles into the conventional matrix.

EDX

**Figure 3:**
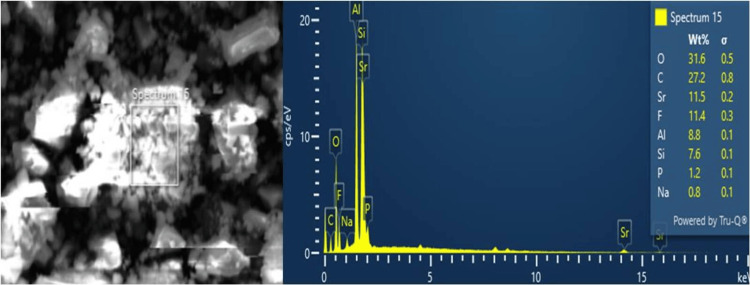
EDX spectrum of nanohydroxyapatite-modified GIC GIC: Glass ionomer cement

The composition of chemicals of the nHA-modified GIC was illustrated (Figure 3).

The graph depicted the presence of various chemical elements, including oxygen (O), calcium (Ca), phosphorus (P), aluminum (Al), sodium (Na), and fluorine (F). Oxygen (O) was the most abundant element detected, comprising 31.6% of the composition. Carbon (C) was found to exist at 27.2%, while fluorine (F) was present at 11.4%. Additionally, aluminum (Al) was identified at 8.8%. Minor elements, such as phosphorus (P) at 1.2% and sodium (Na) at 0.8%, were also recorded through EDX analysis.

SEM

**Figure 4:**
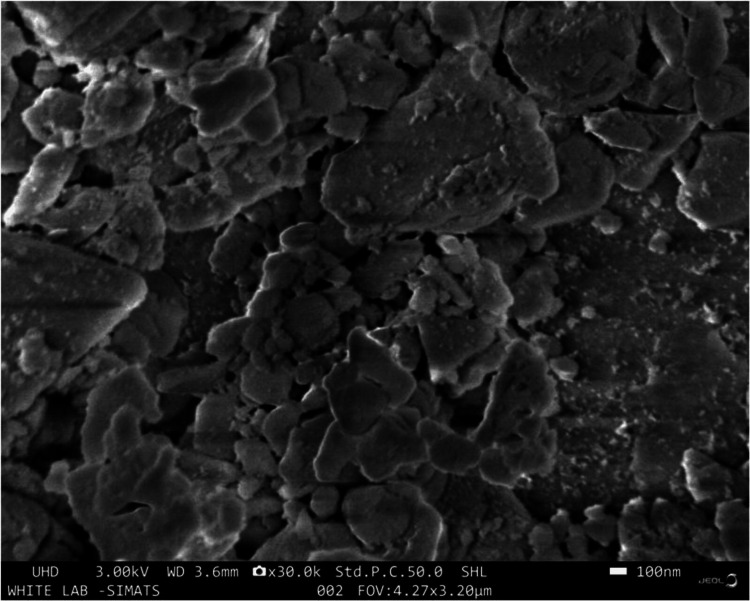
SEM micrograph under magnification of 30,000× for nanohydroxyapatite-modified GIC sample SEM: Scanning electron microscopy; GIC: glass ionomer cement

The SEM images further validate the compacting of hydroxyapatite nanoparticles, resulting in a denser and stronger nHA powder. Examination of the microstructures of the nHA-modified GIC samples reveals noticeable enhancements with subtle alterations to the GIC matrix (Figure 4).

The cement samples exhibited an irregular glass structure shape with uneven particle distribution with a smoother and more compact microstructure, as evident in the SEM images. Spherical and needlelike particles were also detected on the surface of the glass particles. This finding highlights the potential enhancement in mechanical properties and overall performance attributed to the nanomodification of the material.

Antimicrobial activity against *Streptococcus mutans*

**Figure 5:**
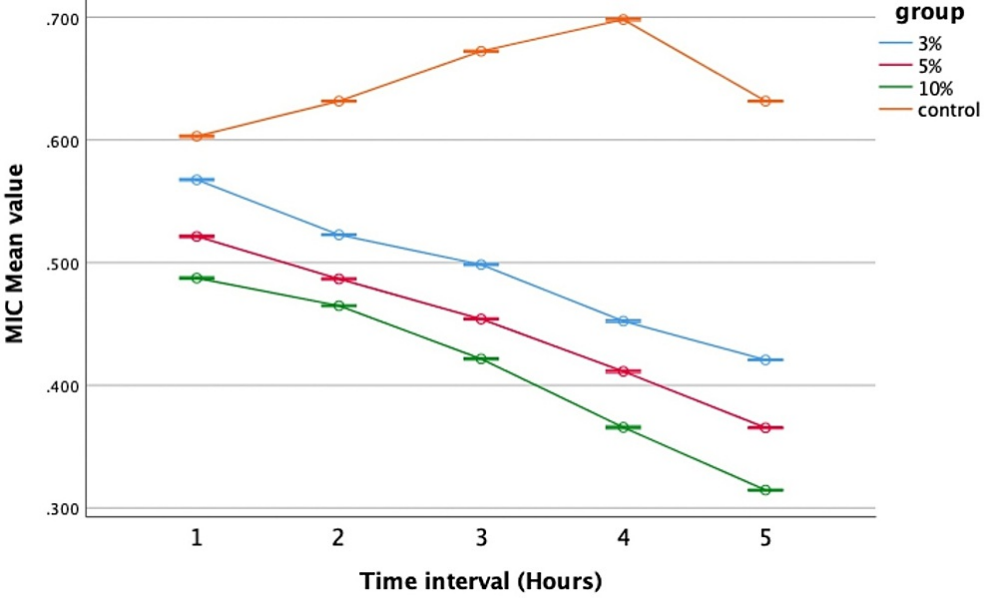
Antimicrobial efficacy against Streptococcus mutans MIC: Minimal inhibitory concentration; error bars: 95% confidence interval The graph illustrates that among the experimental groups, the 10% concentration exhibited the best antimicrobial efficacy, with the lowest mean value recorded particularly at the fifth hour, followed by the 5% and 3% concentrations. In contrast, the control group displayed the highest mean value, indicating the poorest performance. At the end of the fifth hour, the mean value of all experimental groups decreased, with the 10% concentration showing the least reduction

In this investigation, the effectiveness of nHA-modified GIC and conventional GIC against *Streptococcus mutans *was assessed through repeated measures ANOVA. It was observed that the modified groups displayed superior performance against *Streptococcus mutans* compared to the control (Group IV), with significant distinctions noted. Specifically, the 10% concentration (Group III) exhibited robust antimicrobial activity (Figure 5), indicating that a higher weight percentage led to enhanced effectiveness. Analysis via one-way ANOVA indicated significant variations in antibacterial activity against *Streptococcus mutans *across different time intervals, consistently showing the lowest mean values in Group III (0.487, 0.464, 0.421, 0.36, and 0.314 respectively), indicating superior efficacy. Conversely, the control group (Group IV) demonstrated higher mean values (0.603, 0.613, 0.627, 0.689, and 0.631) throughout all time intervals, signifying increased bacterial growth and reduced antibacterial activity compared to the modified GIC groups (Table 1).

**Table 1:** Comparison of minimal inhibitory concentration (MIC) mean value for Streptococcus mutans based on different time intervals *Significant at 0.05, p-value derived from one-way analysis of variance (ANOVA)

_Time interval (hours)_	wt %	N	Mean ± std. deviation	Std. error	95% confidence interval		F value	p-value
					Lower bound	Upper bound		
First	3%	6	0.567 ± 0.00083	0.00034	0.566	0.568	17076.62	0.001*
	5%	6	0.521 ± 0.00051	0.00021	0.521	0.522		
	10%	6	0.487 ± 0.00103	0.00042	0.486	0.488		
	control	6	0.603 ± 0.00126	0.00051	0.601	0.604		
Second	3%	6	0.522 ± 0.00051	0.00021	0.522	0.523	96375.73	0.001*
	5%	6	0.486 ± 0.00051	0.00021	0.486	0.487		
	10%	6	0.464 ± 0.00075	0.00030	0.464	0.466		
	Control	6	0.631 ± 0.00051	0.00021	0.631	0.632		
Third	3%	6	0.498 ± 0.00081	0.00033	0.497	0.499	147332.32	0.001*
	5%	6	0.454 ± 0.00063	0.00025	0.453	0.455		
	10%	6	0.421 ± 0.00054	0.00022	0.421	0.422		
	Control	6	0.672 ± 0.00081	0.00033	0.671	0.673		
Fourth	3%	6	0.452 ± 0.00121	0.00049	0.450	0.453	115857.45	0.001*
	5%	6	0.411 ± 0.00051	0.00021	0.411	0.412		
	10%	6	0.365 ± 0.00103	0.00042	0.364	0.367		
	Control	6	0.698 ± 0.00132	0.00054	0.696	0.699		
Fifth	3%	6	0.420 ± 0.00051	0.00021	0.420	0.421	310621.74	0.001*
	5%	6	0.365 ± 0.00051	0.00021	0.365	0.366		
	10%	6	0.314 ± 0.00083	0.00034	0.314	0.316		
	Control	6	0.631 ± 0.00051	0.00021	0.631	0.632		

Furthermore, Tukey's honestly significant difference (HSD) multiple comparison tests revealed a notable distinction between the control group and the remaining groups (p < 0.05), with the control group (Group IV) demonstrating the lowest efficacy. This comparison also underscored a statistically significant contrast between the control group and the remaining groups, affirming that the nHA-modified groups exhibited increased antimicrobial activity (p < 0.05) in comparison to the control (Table 2).

**Table 2:** Pairwise comparison of antimicrobial efficacy on Streptococcus mutans between all groups *Significant at the 0.05 level; the p-value was derived from multiple comparisons by Tukey's honestly significant difference. The error term is the mean square (error) = 2.250E-7

Pairwise comparison	Mean difference	95% confidence interval		p-value
		Lower bound	Upper bound	
3% vs 5%	0.044	0.043	0.045	0.001*
3% vs 10%	0.081	0.080	0.082	0.001*
3 % vs control	-0.155	-0.155	-0.154	0.001*
5 % vs 10%	0.036	0.036	0.037	0.001*
5 % vs control	-0.199	-0.200	-0.198	0.001*
10 % vs control	-0.236	-0.237	-0.235	0.001*

Antimicrobial activity against *Lactobacillus*

**Figure 6:**
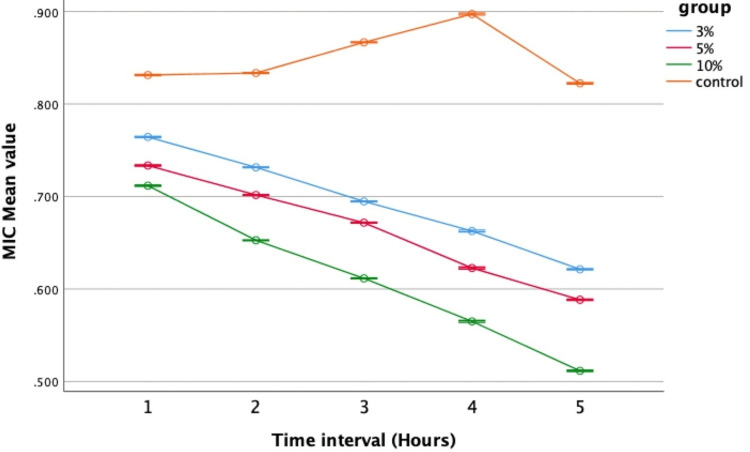
Antimicrobial efficacy against Lactobacillus MIC: Minimal inhibitory concentration; error bars: 95% confidence interval The graph shows that the 10% concentration had the strongest antimicrobial effect, with the lowest mean value observed at the fifth hour, followed by the 5% and 3% concentrations. In contrast, the control group had the highest mean value, indicating the weakest performance

Distinct differences in antimicrobial effectiveness against *Lactobacillus *were evident when comparing the modified groups to the control (Group IV). Analysis via repeated measure ANOVA depicted a linear trend showcasing heightened antibacterial efficacy of nHA-modified GIC groups (Groups I, II, and III) against *Lactobacillus* compared to the control (Figure 6).

Further examination through one-way ANOVA unveiled significant variations across different time intervals against *Lactobacillus,* consistently showing Group III with the lowest mean values of 0.711, 0.65, 0.611, 0.565, and 0.511, respectively, indicating the superior antibacterial potential of the 10% nHA-modified GIC (Table 3).

**Table 3:** Comparison of minimal inhibitory concentration (MIC) mean value for Lactobacillus based on different time intervals *Significant at 0.05; the p-value was derived from one-way analysis of variance (ANOVA)

Time interval (hours)	wt %	N	Mean ± std. deviation	Std. error	95% confidence interval		F value	p-value
					Lower bound	Upper bound		
First	3%	6	0.764 ± 0.00051	0.00021	0.764	0.765	28331.86	0.001*
	5%	6	0.733 ± 0.00054	0.00022	0.733	0.734		
	10%	6	0.711 ± 0.00121	0.00049	0.711	0.714		
	Control	6	0.831 ±0.00051	0.00021	0.831	0.832		
Second	3%	6	0.731 ± 0.00054	0.00022	0.731	0.732	123512.54	0.001*
	5%	6	0.701 ± 0.00051	0.00021	0.701	0.702		
	10%	6	0.652 ± 0.00051	0.00021	0.652	0.653		
	Control	6	0.833 ± 0.00054	0.00022	0.833	0.834		
Third	3%	6	0.694 ± 0.00051	0.00021	0.694	0.695	261426.81	0.001*
	5%	6	0.671 ± 0.00051	0.00021	0.671	0.672		
	10%	6	0.611 ± 0.00054	0.00022	0.611	0.612		
	Control	6	0.866 ± 0.00051	0.00021	0.866	0.867		
Fourth	3%	6	0.662 ± 0.00051	0.00021	0.662	0.663	86487.93	0.001*
	5%	6	0.622 ± 0.00051	0.00021	0.622	0.623		
	10%	6	0.565 ± 0.00089	0.00036	0.564	0.566		
	Control	6	0.897± 0.00213	0.00087	0.894	0.899		
Fifth	3%	6	0.621 ± 0.00081	0.00033	0.621	0.623	156170.43	0.001*
	5%	6	0.588 ± 0.00103	0.00042	0.587	0.589		
	10%	6	0.511 ± 0.00054	0.00022	0.511	0.512		
	Control	6	0.822 ± 0.00081	0.00033	0.821	0.823		

Pairwise comparisons demonstrated notable discrepancies among the control group and the remaining groups (p < 0.05), with the control group (Group IV) exhibiting the least effectiveness, underscoring the enhanced antibacterial capabilities of nHA-modified groups (p < 0.05) (Table 4).

**Table 4:** Pairwise comparison of antimicrobial efficacy on Lactobacillus between all groups *Significant at the 0.05 level; the p-value was derived from multiple comparisons by Tukey's honestly significant difference. The error term is the mean square (error) = 1.273E-7

Pairwise comparison	Mean difference	95% confidence interval		p-value
		Lower bound	Upper bound	
3% vs 5%	0.031	0.030	0.031	0.001*
3% vs 10%	0.084	0.083	0.085	0.001*
3% vs control	-0.155	-0.155	-0.154	0.001*
5% vs 10%	0.053	0.052	0.053	0.001*
5% vs control	-0.186	-0.187	-0.186	0.001*
10% vs control	-0.239	-0.240	-0.239	0.001*

Compressive strength

**Figure 7:**
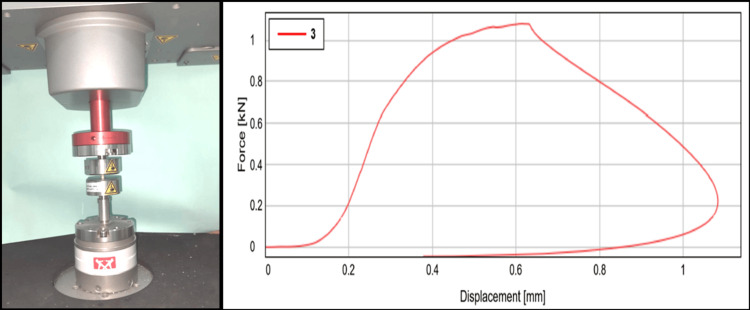
Compressive strength testing The figure depicts the samples loaded for testing  in a universal testing machine and the linear graph of compressive strength of nanohydroxyapatite-modified glass ionomer cement

The specimens underwent compression testing, and the values were graphed linearly (Figure 7).

An ANOVA analysis showed a notable difference in compressive strength across the groups, with an F value of 770.02 and a p-value of 0.001 (p < 0.05) (Table 5).

**Table 5:** Comparison between groups for the evaluation of compressive strength *Significant at 0.05; the p-value was derived by one-way analysis of variance (ANOVA)

wt%	N	Mean ± std. deviation	Std. error	95% confidence interval		F value	p-value
				Lower bound	Upper bound		
3%	12	172.55 ± 0.769	0.222	172.06	173.04	770.02	0.001*
5%	12	178.16 ± 0.760	0.219	177.68	178.65		
10%	12	182.45 ± 0.950	0.274	181.85	183.06		
Control	12	162.46 ± 1.606	0.463	161.44	163.48		

Tukey's posthoc test revealed a significant difference in performance, indicating that the modified groups surpassed the control group (p < 0.05). Moreover, a notable distinction was noted between Group III and the remaining groups (p < 0.05) indicating that Group III, with higher concentrations, displayed superior compressive strength compared to the others (Table 6).

**Table 6:** Pairwise comparison for the evaluation of compressive strength *Significant at 0.05; the p-value was derived from Tukey's posthoc test

Pairwise comparison	Mean difference	95% confidence interval		p-value
		Lower bound	Upper bound	
3% vs 5%	-5.608	-6.784	-4.432	0.001*
3% vs 10%	-9.900	-11.075	-8.724	0.001*
3% vs control	10.091	8.915	11.267	0.001*
5% vs 10%	-4.291	-5.467	-3.115	0.001*
5% vs control	15.700	14.524	16.875	0.001*
10 % vs control	19.991	18.815	21.167	0.001*

## Discussion

There is an ongoing quest for materials that can effectively adhere to the tooth structure to minimize the risk of restoration failure. In recent years, there has been a growing focus on discovering and developing new dental materials as well as modifying existing ones by incorporating beneficial additives. The GIC is renowned for its capacity to chemically adhere to the tooth structure. However, its mechanical shortcomings, such as limited compressive strength, fracture resistance, toughness, and wear resistance, have limited its usage [19]. To address these shortcomings, modifying conventional GIC is essential. It's crucial that such modifications do not compromise the original material's physical properties and bonding ability. Therefore, the incorporation of hydroxyapatite nanoparticles into the GIC through green synthesis, characterization, and antimicrobial evaluation presents a complex yet significant advancement with implications for both material science and dentistry.

Bacteria found in the oral cavity are significant contributors to the development of dental caries. Numerous studies have established a direct correlation between elevated levels of *Streptococcus mutans *bacteria and increased dental caries incidence. Consequently, it is imperative for the GIC to possess potent antibacterial properties to prevent dental caries and ensure long-lasting restorations [20]. In our study, the antimicrobial efficacy was markedly enhanced in groups modified with nHA. This result aligns with the research conducted by Alatawi et al. [21], who assessed the antimicrobial activity of the GIC by incorporating various concentrations of nHA. They observed that an increase in the weight percentage of nHA up to 10% resulted in enhanced antimicrobial activity. The inhibition zones in this study can be attributed to nanoparticles penetrating bacterial cells and inducing oxidative stress, thereby inhibiting growth and causing bacterial cell death [22]. Similarly, Shinonaga et al. [23] examined the antimicrobial effects of the GIC incorporated with hydroxyapatite and discovered that the presence of hydroxyapatite improved its antibacterial properties compared to traditional GIC samples. Pagano et al. [24] stated that incorporating 4% nHA into the GIC improved its antibacterial activity against *Streptococcus mutans* bacteria, corroborating our results. Additionally, Zaki et al. [25] conducted a study wherein no failures due to secondary caries might be due to the cariostatic action of the GICs caused by the sustained release of fluoride over time.

Gupta et al. [26] observed that incorporating hydroxyapatite into the GIC led to improved matrix strength and enhanced bonding between the glass core and matrix. This enhancement is attributed to apatite formation and ion release from glass ionomer, which contribute to the physical characteristics of the cement. Barandehfard et al. [27] studied the integration of nHA into the GIC and observed that the modified GIC demonstrated notably greater compressive strength in comparison to the unmodified sample. The enhanced compressive strength of the GIC with hydroxyapatite nanoparticles is attributed to increased crystallinity and the presence of nanoceramics. Moheet et al. [28] assessed the compressive strength of the GIC supplemented with nHA-silica particles and found that this modification increased compressive strength compared to conventional GIC by filling void spaces and preventing crack formation. Moshaverinia et al. [29] illustrated that integrating 2% hydroxyapatite nanoparticles into the GIC improved its physical and mechanical properties. Additionally, Sajjad et al. [30] found that incorporating nano-ZrO2-SiO2-HA significantly enhanced the compressive strength of conventional GIC. These findings align with our results, indicating that the addition of hydroxyapatite nanoparticles yields promising enhancements in GIC properties.

The findings revealed that GICs containing various weight percentages of HA exhibited significant antimicrobial activity and higher compressive strength. This improvement can be ascribed to the influence of nHA on the formation of polysalt bridges within each GIC and its setting reaction, thereby enhancing the characteristics of the resulting material. The increase in mechanical strength is facilitated by chemical transformations occurring during the early solidification of GICs incorporating HA. Hydroxyapatite, being particulate, undergoes dissolution in acidic conditions, with its solubility significantly rising upon contact with polycarboxylic acid. This leads to the release of calcium ions from the surface of HA, enhancing the acid-base reaction and introducing salt bridges into the GIC structure, thus forming cross-links and ultimately leading to a stronger cement. Moreover, the incorporation of HA into the GICs has been observed to enhance their density by efficiently filling the interstitial spaces between glass particles within the GIC matrix. Additionally, due to their expansive surface area, hydroxyapatite nanoparticles can interact with microorganisms through electrostatic attraction. Positively charged hydroxyapatite nanoparticles possess the ability to adhere to negatively charged surfaces, such as those of bacteria and other pathogens, thereby disrupting their cell membranes and inhibiting their growth.

Furthermore, the study incorporates a green synthesis of nHA to mitigate cytotoxicity, aligning with sustainability and environmental responsibility values in the production of hydroxyapatite nanoparticles for GIC application. This approach typically employs green solvents, energy-efficient techniques, and environmentally friendly precursors to minimize adverse environmental impacts and the use of hazardous chemicals. The decision to incorporate nanoparticles into the GIC matrix was motivated by their ability to improve dispersion, thereby enhancing the performance of conventional GIC. This enhancement is attributed to the reduction of pores within the matrix, leading to a more effective increase in packing density. Thus, by preventing microbial adhesion to tooth surfaces, hydroxyapatite nanoparticles can reduce biofilm formation, a precursor to dental plaque and cavities. The release of calcium ions from hydroxyapatite nanoparticles disrupts bacterial growth and may induce bacterial death by disrupting cell membranes. The combination of the GIC's excellent tooth adhesion, the antibacterial properties of hydroxyapatite, and high compressive strength results in a dental material suitable for both restorative and preventive purposes, particularly in restorative dentistry. The research findings indicate that incorporating nHA into conventional GIC improves its resistance to compression and its ability to inhibit the growth of *Streptococcus mutans *and *Lactobacillus*. Additionally, conventional GIC and nHA underwent FTIR, EDX, and SEM analysis to confirm the successful nanoparticle loading process. This indicates that the incorporation of nHA particles into the GIC holds promise as a means to enhance mechanical properties and suppress residual bacteria within dentin. However, it is important to note certain limitations in this study. Factors such as normal masticatory stress, moisture, intrapulpal pressure, and operator variability within the oral cavity were not accounted for. Therefore, additional in vivo investigations including molecular chemistry and bonding mechanisms to be studied to evaluate the long-term durability of these materials.

## Conclusions

Therefore, the addition of green-synthesized nHA into the GIC resulted in significant enhancements in both antibacterial efficacy and compressive strength. This integration of green-synthesized nHA into the GIC demonstrates substantial improvements in its properties, rendering it advantageous for restorative purposes, especially in high-stress areas, as a potential replacement for conventional commercial GIC. Further research should address intraoral factors like normal masticatory stress, moisture, and intrapulpal pressure.
